# Probucol dramatically enhances dihydroartemisinin effect in murine malaria

**DOI:** 10.1186/s12936-016-1532-y

**Published:** 2016-09-15

**Authors:** Aiko Kume, Dang Trinh Minh Anh, Mototada Shichiri, Noriko Ishida, Hiroshi Suzuki

**Affiliations:** 1 Research Unit for Functional Genomics, National Research Center for Protozoan Diseases, Obihiro University of Agriculture and Veterinary Medicine, Obihiro City, Hokkaido Japan; 2 The United Graduate School of Veterinary Sciences, Gifu University, Gifu City, Japan; 3 Microbiology and Immunology Department, Pasteur Institute in Ho Chi Minh City, Ministry of Health, Ho Chi Minh City, Vietnam; 4 Biomedical Research Institute, National Institute of Advanced Industrial Science and Technology (AIST), Ikeda City, Osaka Japan

**Keywords:** C57BL/6 J mice, Dihydroartemisinin, Malaria, Oxidative stress, Probucol, *Plasmodium yoelii* XL-17, Vitamin E

## Abstract

**Background:**

Artemisinin-based combination therapy (ACT) has been adopted as national policy for the first-line treatment in large number of malaria-endemic regions. However, artemisinin-resistant parasites have emerged and are spreading, with slow-cleaning parasites being reported in patients treated with ACT. It means that more parasites are exposed to the partner drug alone and the risk of developing resistant parasites against the partner drug is increasing. Therefore, the development of a new method to enhance the effect of artemisinin is required. In this study, the potential effect of probucol as a combination drug of dihydroartemisinin (DHA), an artemisinin derivative, was examined.

**Methods:**

C57BL/6 J mice infected with *Plasmodium yoelii* XL-17 were treated with probucol and/or DHA. The mice were fed with a probucol mixed diet from 2 weeks before infection and through infection period. DHA was injected to mice three to 5 days post infection once a day. In addition, 0.5 % (w/w) probucol was mixed with vitamin E supplemented diet (800 mg/kg) and fed to mice infected with *P. yoelii* XL-17 to examine the mechanisms of probucol on murine malaria. Furthermore, 8-OHdG, a biomarker of oxidized DNA, was detected in infected red blood cells (iRBC) taken from infected mice by immunofluorescent staining.

**Results:**

With dose-dependent manner, both probucol and DHA decreased parasitaemia and increased survival rate of mice infected with *P. yoelii* XL-17. A significantly larger amount of 8-OHdG was detected in iRBC taking from probucol-treated mice than control mice. In addition, a large amount of vitamin E supplementation eliminated the effect of probucol against *P. yoelii* XL-17 infection and lowered the effect of probucol on host plasma vitamin E concentration. The effective doses for probucol and DHA were 0.5 % and 30 mg/kg, respectively, in each single treatment. While the combination treatment of 0.25 % probucol and 7.5 mg/kg DHA was effective in all mice from *P. yoelii* XL-17 infection.

**Conclusion:**

This study demonstrated that probucol has some impact on malaria by oxidative stress through the induction of host plasma vitamin E deficiency. Moreover, the effective dose of DHA on malaria was decreased by prophylactic treatment of probucol. This finding indicates that probucol might be a candidate for a prophylactic treatment drug to enhance the effect of DHA.

## Background

Every year more than 125 million international travellers visit over 100 tropical and sub-tropical countries and territories where malaria is endemic. Many travellers from malaria-free areas fall sick with malaria during visits to endemic areas and each year over 10,000 cases are reported to develop malaria after returning home [1, 2].

Current treatment and prevention methods against malaria are chemoprophylactic anti-malarial drugs and vector controls. Artemisinin and its derivatives have become essential components of anti-malarial treatment and their rapid clinical and parasitological responses are life saving in severe malaria [3]. However, these features invoke a potential problem of inadequate treatment because malaria patients who quickly feel better after taking these drugs tend to stop their treatment before completion of the regime. Such cessation provides the risk of emerging artemisinin-resistant parasites [4, 5]. The emergence of resistant parasites may also be caused by artemisinin monotherapy, as failure to complete treatment and the re-appearance of parasites have occasionally been observed among patients who were treated with artemisinin monotherapy [5, 6]. In 2014, drug efficacy studies have detected artemisinin-resistant *Plasmodium falciparum* in Cambodia, the Lao People’s Democratic Republic, Myanmar, Thailand, and Vietnam [7]. The first artemisinin resistance was reported in western Cambodia [8] and the rate of slow-clearing infections rapidly increased from 0.6 % in 2001 to 20 % in 2010 in western Cambodia [9].

Drug combinations are effective in delaying or preventing development of drug resistance [10]. Artemisinin-based combination therapy (ACT), a combination chemotherapy of artemisinin derivative and other anti-malarial drugs, is the most effective treatment for malaria today. World Health Organization (WHO) recommends ACT as the first-line treatment for uncomplicated falciparum malaria. Patients with vivax malaria should also be treated with appropriate ACT in areas where resistance to chloroquine has been documented [7]. In 2013, ACT had been adopted as national policy for first-line treatment in 79 of 87 countries where *P. falciparum* is endemic [7]. Despite the recent spread of artemisinin-resistant parasites, ACT is still highly efficacious in these regions, presumably, due to increased reliance on the efficacy of the partner drugs as the potency of the artemisinin component declined [11]. However, slow-cleaning parasites in patients treated with ACT causes more parasites to be exposed to the partner drug alone, increasing the risk of developing resistance to the partner drug [12]. Therefore, the development of a new method to enhance the effect of artemisinin is required.

Numerous studies have demonstrated the relationship between host nutrition status and malaria morbidity and mortality. A systematic review indicates that apparent malnutrition does not have great impact on malaria morbidity, but could have a negative impact on malaria mortality and severity [13]. Not only malnutrition but also deficiency of micronutrients, such as iron, zinc and vitamin A, showed a positive or negative effect on malaria [14, 15]. Previous studies revealed that the modification of host nutritional status to lowering vitamin E concentration conferred resistance to malarial infection [16, 17]. Recently, probucol, an antihyperlipidaemia drug, has been shown to reduce the plasma concentration of α-tocopherol, a type of vitamin E, by inhibiting ATP-binding transporter A1 [18], and to enhance the host resistance against malaria in mice [19, 20].

In this study, to ascertain whether probucol is appropriate to enhance the effect of dihydroartemisinin (DHA), an artemisinin derivative, C57BL/6 J mice were treated with probucol and DHA, and infected with *Plasmodium yoelii* XL-17, a lethal strain of rodent malaria. In addition, the relationship between the effect of probucol on murine malaria and on host plasma vitamin E concentration was examined.

## Methods

### Mice

C57BL/6 J mice were bred and maintained in specific pathogen-free conditions at the National Research Center for Protozoan Diseases, Obihiro University of Agriculture and Veterinary Medicine, Japan. Male mice aged eight to ten weeks were used in this study. The room temperature (24 ± 1 °C) and humidity (50 ± 10 %) were regulated and lighting was controlled (lights on from 07:00 to 19:00). Mice had free access to water and commercial regular diet containing 80 mg vitamin E/kg (CA-1; CLEA Japan, Tokyo, Japan). All the animal experiments in this paper were conducted in accordance with the standards relating to the Care and Management of Experimental Animals of Obihiro University of Agriculture and Veterinary Medicine, Japan.

### Effect of probucol on *Plasmodium yoelii* XL-17 infection in mice

With commercial regular diet, were mixed 0.1, 0.25 and 0.5 % (w/w) probucol (4,4′-[(1-Methylethylidene)bis(thio)]bis[2,6-bis(1,1-dimethylethyl)phenol], 168-20313, Wako, Osaka, Japan) and fed on C57BL/6 J mice for 2 weeks. Then, 4 × 10^4^*P. yoelii* XL-17 infected red blood cells (iRBC) were intraperitoneally inoculated in both probucol-treated mice and control mice fed with commercial diet (day 0). Probucol treatment was continued throughout the infection period. Survival rates and parasitaemia were monitored in all experimental groups.

Blood smears were prepared using blood collected from mice fed with 0.5 % (w/w) probucol-mixed diet or commercial regular diet and subsequently infected with *P. yoelii* XL-17, when their parasitaemia ranged from 20 to 30 %. The smears were fixed with methanol/acetone (1:1 in volume) at −30 °C for 10 min. After washing with PBS three times, an antibody solution was added on the blood smear and incubated at 4 °C overnight. The antibody solution was prepared as follows [21], 1 μg/ml of anti-8-OHdG monoclonal antibody (N45.1; MOG-020P, Japan Institute for the Control of Aging, NIKKEN SEIL Co., Ltd, Shizuoka, Japan) and 0.1 % (v/v) Alexa FluorR 488 goat anti-mouse IgG(H + L) (A-11001, Invitrogen, Carlsbad, CA, USA) were dissolved in 5 % skimmed milk in PBS and incubated at 4 °C overnight. Then, mouse serum inactivated by incubation at 56 °C for 30 min was added to the solution and incubated at 4 °C for 2 h. After treatment with antibody solution, the smears were washed with PBS three times and incubated with Hoechst 33342 (346-07951, DOJINDO, Kumamoto, Japan) at 2 μg/ml diluted with PBS at 37 °C for 30 min. After washing with PBS, the smears were mounted with glycerol. Fluorescent signal was detected by a fluorescent microscope (BZ-9000, KEYENCE, Osaka, Japan). Fluorescence intensity of anti-8-OHdG signal was measured by ImageJ [22].

### Effect of probucol on *Plasmodium yoelii* XL-17 infection in mice fed with vitamin E supplemented diet

C57BL/6 J males were treated with 0.5 % (w/w) probucol mixed with 800 mg/kg vitamin E supplemented diet (CE-2 based vitamin E supplemented diet; CLEA Japan, Tokyo, Japan) for 2 weeks. Blood samples were treated with EDTA for anticoagulation and centrifuged (5000 rpm, 5 min, 4 °C) to obtain plasma. Plasma α-tocopherol was extracted using a protocol described previously [23]. Briefly, the samples were treated with chloroform/methanol (2:1 in volume) containing 100 µM butylated hydroxytoluene. Thereafter, the extracts of these samples were centrifuged at 15,000 rpm at 4 °C. The concentration of α-tocopherol was determined by using an HPLC-ECD system with an electrochemical detector (NANOSPACE SI-1, Shiseido, Tokyo, Japan). The analytes were eluted with methanol containing 50 mM NaClO_4_ at a flow rate of 0.7 mL/min in a Wakosil-2 5C18 RS column (Wako, Tokyo, Japan). To determine the concentration of α-tocopherol, the area under the curve of the analyte was compared with that of the standard. The standard curve was prepared by using serial dilutions (1 µM, 500 nM, and 100 nM) of α-tocopherol standard (Eisai Chemical Company, Tokyo, Japan).

*Plasmodium yoelii* XL-17 iRBC (4 × 10^4^ iRBC/head) were inoculated by intraperitoneal injection to the probucol-treated mice fed with vitamin E supplemented diet and control mice fed with commercial regular diet (CE-2; CLEA Japan, Tokyo, Japan) (day 0). Probucol treatment was continued through infection period. Survival rates and parasitaemia were monitored in all experimental groups.

### Effect of DHA on *Plasmodium yoelii* XL-17 infection in mice

C57BL/6 J male mice were inoculated with 4 × 10^4^*P. yoelii* XL-17 iRBCs by intraperitoneal injection (day 0). From three to 5 days post infection (dpi), 7.5, 15 and 30 mg/kg of DHA (D3793, Tokyo Chemical Industry, Tokyo, Japan) or[of?] solvent were injected to infected mice by intraperitoneal injection once a day. DHA was formulated in 50 % (v/v) dimethyl sulfoxide (D5879, Sigma-Aldrich, Mo, USA) in Polyoxyethylene (20) Sorbitan Monooleate (161-21621, Wako, Osaka, Japan), with a final injection volume of 100 μl. Survival rates and parasitaemia were monitored in all experimental groups.

### Combination effect of probucol and DHA on *Plasmodium yoelii* XL-17 infection in mice

Mice were separated into four groups, solvent treatment as a control group (Solvent), 0.25 % (w/w) probucol treatment group (Pro), 7.5 mg/kg DHA treatment group (DHA), and 0.25 % (w/w) probucol and 7.5 mg/kg DHA combination treatment group (Pro + DHA). In both Pro and Pro + DHA groups, mice were treated with probucol from 2 weeks before infection and throughout infection period. Solvent and DHA groups mice were fed with commercial regular diet throughout experiment period. *Plasmodium yoelii* XL-17 iRBCs (4 × 10^4^ iRBC/head) were inoculated to mice by intraperitoneal injection (day 0). On 3–5 dpi, 7.5 mg/kg of DHA was injected to DHA and Pro + DHA groups mice by intraperitoneal injection once a day. Solvent group mice were injected with solvent on 3–5 dpi once a day. Survival rates, parasitaemia and haemoglobin concentration were monitored in all experimental groups. Haemoglobin concentration was measured by Celltac α (MEK-6450, Nihon Kohden Corp., Tokyo, Japan).

### Statistical analysis

Statistical analyses were performed by Student *t* test with Excel. The survival rate was analysed by using the Log-rank (Mantel-Cox) Test and Gehan-Breslow-Wilcoxon Test with GraphPad Prism 5. For all analyses, a p-value less than 0.05 was considered statistically significant.

## Results

### Effect of probucol on *Plasmodium yoelii* XL-17 infection in mice

**Fig. 1:**
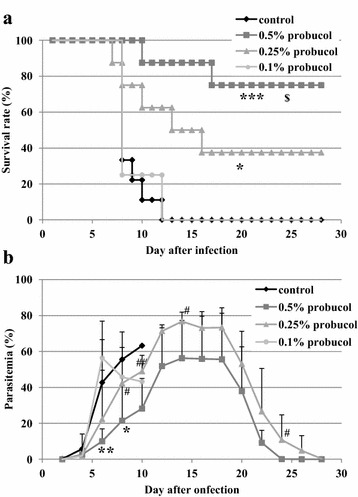
Effect of probucol on *Plasmodium yoelii* XL-17 infection in mice. C57BL/6 J mice were fed with 0.1 % (w/w), 0.25 % (w/w) and 0.5 % (w/w) probucol mixed diet from 2 weeks before infection to through infection period. *Plasmodium. yoelii* XL17 (4 × 10^4^ iRBC/head) were inoculated into drug-treated mice and control mice fed with regular commercial diet by intraperitoneal injection. Survival rate (**a**) and parasitaemia (**b**) were monitored for 28 days post infection. Control, n = 9; 0.5 % probucol, n = 8; 0.25 % probucol, n = 8; 0.1 % probucol, n = 4. The data presented are mean ± SD. Compared with control mice: *p < 0.05, **p < 0.005, ***p < 0.0005. Compared with 0.5 % probucol-treated mice:, ^#^ *p* < 0.1, ^##^ *p* < 0.05. Compared with 0.1 % probucol-treated mice: ^$^ *p* < 0.005

**Fig. 2:**
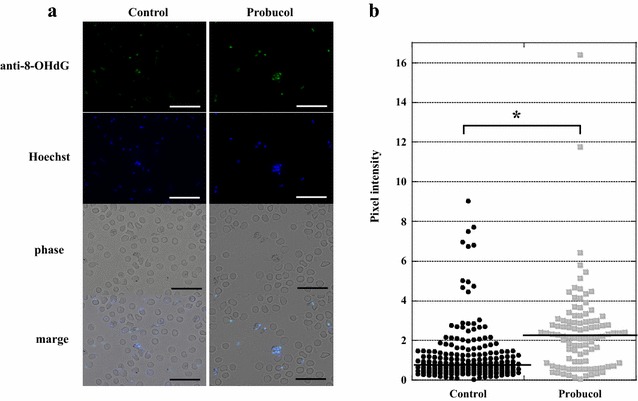
Detection of 8-OHdG in *Plasmodium yoelii* XL-17 infected RBC of probocol treated mice. 8-OHdG in iRBC was detected by immunofluorescence staining and observed by a fluorescent microscope (**a**). The blood smears were made from mice fed with 0.5 % (w/w) probucol mixed diet or normal diet and infected with *P. yoelii* XL-17 when their parasitaemia were around 20–30 %. The *line* shows the median. The fluorescence intensity of anti-8-OHdG signal was measured by ImageJ (**b**). *Scale bar* is 20 μm. *p < 1×10^−8^

All of 0.1 % probucol-treated mice and control mice died within 12 dpi. On the other hand, the survival rates of 0.25 and 0.5 % probucol-treated mice were 38 and 75 %, respectively, on 28 dpi (Fig. 1a). 0.25 and 0.5 % probucol treatment significantly increased survival rates as compared with control (*p* < 0.05). The survival rate of 0.5 % probucol treatment were significantly higher than that of 0.1 % probucol treatment (*p* < 0.005). On 6 and 8 dpi, 0.5 % probucol-treated mice showed significantly lower parasitaemia than control mice (*p* < 0.05), while 0.1 and 0.25 % treated mice did not show any significant difference in parasitaemia (Fig. 1b). Compared with 0.25 % probucol treatment, the 0.5 % probucol-treated mice showed lower parasitaemia throughout the experiment (Fig. 1b). 8-OHdG was detected in iRBCs taken from both control and 0.5 % probucol-treated mice (Fig. 2a). Although the fluorescence intensity of iRBCs had a wide range distribution in both control and probucol-treated mice, the mean intensity in probucol-treated mice (mean ± SD = 2.47 ± 2.22) was significantly higher than that in control mice (1.22 ± 1.41; *p* < 1×10^−8^) (Fig. 2b).

### Effect of probucol on *Plasmodium yoelii* XL-17 infection in mice fed with vitamin E supplemented diet

**Fig. 3:**
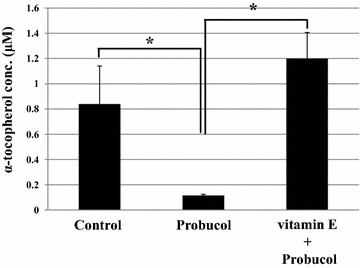
Effect of probucol treatment with vitamin E on plasma α-tocopherol concentration in mice. C57BL/6 J males were fed with 0.5 % (w/w) probucol mixed diet or 0.5 % probucol and 800 mg/kg vitamin E mixed diet for 2 weeks and subsequently their blood was taken. Control mice were fed with regular commercial diet. The data presented are mean ± SD. Control: n = 9; probucol: n = 5; vitamin E +probucol: n = 4, *p < 0.0005

**Fig. 4:**
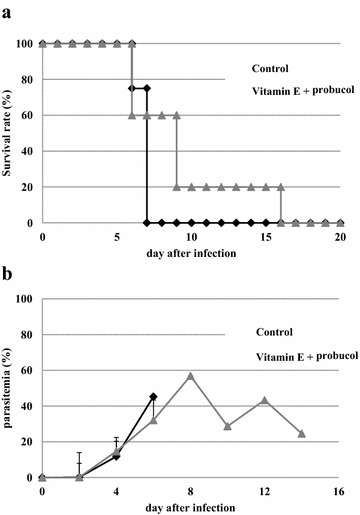
Effect of probucol treatment with vitamin E supplemented diet on *Plasmodium yoelii* XL-17 infection in mice. C57BL/6 J males were fed with 0.5 % (w/w) probucol mixed and 800 mg/kg vitamin E supplemented diet from 2 weeks before infection to throughout infection period. Survival rate (**a**) and parasitaemia (**b**) were monitored. *P. yoelii* XL-17 (4 × 10^4^ iRBC/head) were inoculated in drug treated mice and control mice fed with regular commercial diet by intraperitoneal injection. The data presented mean ± SD. Five mice were used for each group and the experiment was duplicated

Probucol treatment significantly decreased plasma α-tocopherol concentration, while probucol-treated mice fed with vitamin E supplemented diet showed higher α-tocopherol concentration than that of probucol-treated mice fed with regular diet (Fig. 3). There was no significant difference in α-tocopherol concentration between control and probucol-treated mice fed with vitamin E supplemented diet. After the *P. yoelii* XL-17 infection, all control mice died by 7 dpi. The survival rate of 0.5 % probucol treatment in mice fed with vitamin E supplemented diet was 20 % on 9 dpi and it decreased to 0 % on 16 dpi (Fig. 4a). There were no significant differences between experimental groups on both survival rate and parasitaemia (Fig. 4a, b).

### Effect of DHA on *Plasmodium yoelii* XL-17 infection in mice

**Fig. 5:**
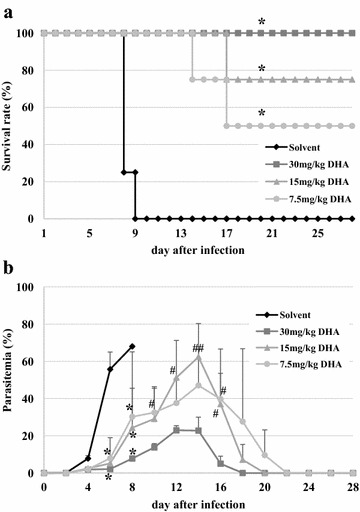
Effect of DHA on *Plasmodium yoelii* XL-17 infection in mice. *Plasmodium yoelii* XL17 (4 × 10^4^ iRBC/head) were inoculated to C57BL/6 J males fed with regular commercial diet by intraperitoneal injection. The mice were treated with 7.5, 15 and 30 mg/kg of DHA by intraperitoneal injection on 3 to 5 days post infection once a day. Solvent was injected to mice as control. Survival rate (**a**) and parasitaemia (**b**) were monitored. Four mice were used for each group. The data are mean ± SD. DHA mice compared with mice injected with solvent: *p < 0.01. DHA 7.5, 15 mg/kg compared with 30 mg/kg DHA treated mice: ^#^ *p* < 0.05, ^##^ *p* < 0.01

As shown in Fig. 5a, the survival rates of all of DHA-treated groups were significantly higher than that of control (*p* < 0.01). There were no significant differences among experimental groups in survival rate. In addition, all DHA-treated mice showed significantly lower parasitaemia than that of control mice on 6 and 8 dpi (*p* < 0.01) (Fig. 5b). On 12, 14 and 16 dpi, 30 mg/kg DHA-treated mice showed significantly lower parasitaemia than 15 mg/kg DHA-treated mice (*p* < 0.05). Moreover, 30 mg/kg DHA-treated mice showed significantly lower parasitaemia than 7.5 mg/kg DHA-treated mice on 10 and 16 dpi (*p* < 0.05). Compared with 15 mg/kg DHA treatment, the 7.5 mg/kg DHA-treated mice did not show any significant difference in parasitaemia throughout the experiment.

### Combined effect of probucol and DHA on *Plasmodium yoelii* XL-17 infection in mice

**Fig. 6:**
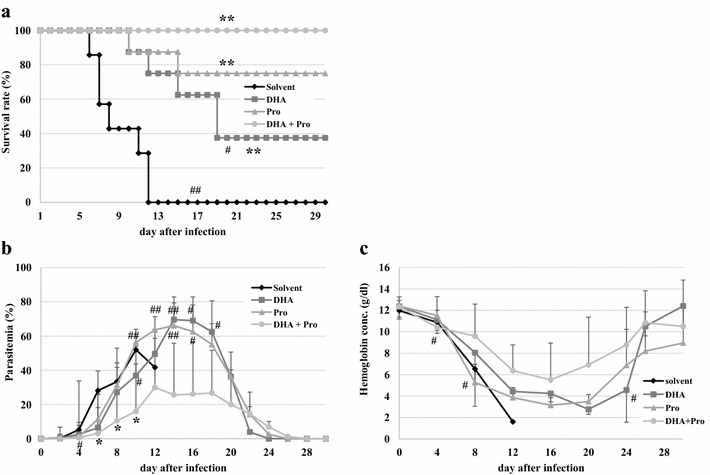
Effect of combination of DHA and probucol on *Plasmodium yoelii* XL-17 infection in mice. Probucol only group (Pro, n = 8) and probucol and DHA combination group (Pro + DHA, n = 8) were fed with 0.25 % (w/w) probucol mixed diet from 2 weeks before infection to throughout infection period. DHA group (DHA, n = 8) and Pro + DHA group were treated with 7.5 mg/kg of DHA by intraperitoneal injection on 3–5 days post infection once a day. *Plasmodium yoelii* XL-17 (4 × 10^4^ iRBC/head) were inoculated in mice by intraperitoneal injection. Solvent was injected to mice as control (n = 7). Survival rate (**a**), parasitaemia (**b**) and haemoglobin concentration (**c**) were monitored. The data are mean ± SD. Treated group compared with Solvent group: *p < 0.05, **p < 0.01. Single treatment groups compared with Pro + DHA group: ^#^ *p* < 0.05, ^##^ *p* < 0.01

The survival rates of solvent control, 7.5 mg/kg DHA, 0.25 % probucol and combination of 0.25 % probucol and 7.5 mg/kg DHA-treated mice were 0, 38, 75, and 100 %, respectively, on 19 dpi (Fig. 6a). All drug treatment groups significantly increased mouse survivals (*p* < 0.01). Survival rate of combined treatment was significantly higher than that of DHA alone (*p* < 0.05). Combined treatment showed significantly lower parasitaemia compared with solvent control on 6, 8 and 10 dpi (*p* < 0.05). Parasitaemia of both probucol single and DHA single treatment mice were significantly higher than that of combination treatment mice on 4, 10, 12 and 14 dpi (*p* < 0.05) (Fig. 6b). In addition, peak parasitaemia of both probucol- and DHA-treated mice were around 70 %, respectively, on 14 dpi, whereas that of probucol and DHA combined mice was about 30 % on 12 dpi. Haemoglobin concentration of combination treatment mice was significantly higher than probucol single treatment mice on 8 and 12 dpi (*p* < 0.05) (Fig. 6c). Furthermore, combined treatment showed significantly higher haemoglobin concentration than DHA single treatment on 24 dpi.

## Discussion

The results of this study showed that the effective dosage of probucol and DHA were 0.5 % (w/w) and 30 mg/kg, respectively, when mice were infected with 4 × 10^4^ of *P. yoelii* XL-17 iRBCs (Figs. 1, 5). However, treatment combined with lower dosages of both drugs, 0.25 % probucol and 7.5 mg/kg DHA, saved all mice from *P. yoelii* XL-17 infection (Fig. 6). The peak of parasitaemia in mice treated with combined drugs at lower dosage was equivalent to higher dosage of DHA (30 mg/kg) single treated mice (Figs. 5, 6). In addition, the timing of decreasing parasitaemia in mice treated with combined drugs was also similar to that in mice treated with high dose DHA single treatment (Figs. 5, 6). These results indicate that probucol prophylactic treatment enhances the effect of DHA on *P. yoelii* XL-17 infection in mice.

Previously, the lowering effect of probucol treatment on plasma vitamin E concentration, has been estimated [19, 20]. In this study, supplementation of vitamin E dramatically eliminated the resistant effect of probucol against *P. yoelii* XL-17 infection and simultaneously negated the lowering effect of probucol on host plasma vitamin E concentration (Figs. 3, 4). In addition, significantly a larger amount of 8-OHdG, a biomarker of oxidized DNA, was detected in iRBCs taken from probucol-treated mice than those from control mice (Fig. 2). These results indicate that malaria parasites infecting probucol-treated mice were damaged by oxidative stress. Probucol impacts on rodent malaria by oxidative stress through the induction of host plasma vitamin E deficiency. In previous studies, it has been shown that dietary or genetically induced vitamin E deficiency enhances oxidized products in parasites and malaria resistant phenotype in host [17, 24]. Vitamin E, which has potent anti-oxidative activity, may be utilized by parasites for their survival and its deficiency may induce oxidative stressful environment around parasites. With increasing dosage of DHA, which directly targets parasites [25, 26], peak of parasitaemia was controlled to approximately 20 % after infection (Fig. 5). On the other hand, probucol treatment in spite of higher dosage showed elevated peak of parasitaemia (60 %), although mortality of mice was low after the infection (Fig. 1). In addition, the effect of probucol with higher dosage (0.5 %) disappeared when mice were fed with significant amount of vitamin E along with the probucol treatment (Fig. 4). Taken together, these results suggest that probucol might inhibit parasite proliferation and survival through vitamin E deficiency. Hence, probucol has an indirect effect on parasites; this suggests that the development of resistance to probucol among *Plasmodium* parasites is logically unlikely. Probucol single and combination treatment did not clear parasitaemia in the mice, however it increased mouse survival. These treatments might slow down parasite growth and/or development of the immune system to control the parasitaemia. In a previous study, α-tocopherol deficiency caused by inhibition of α-tocopherol transfer protein enhanced the acquired immune response in murine malaria infection [27].

Artemisinin is an extract of *Artemisia annua*, a traditional Chinese herbal medicine. Its anti-malarial properties were discovered in 1971 [3]. ACT is used as first-line treatment all over the world today [7]. The absorbed artemisinin derivatives are converted primarily to DHA and metabolized to inactive form via hepatic cytochrome P-450 and other enzyme systems [28, 29]. Electrochemical studies showed that haem/iron catalyzes the irreversible breakdown of artemisinin derivatives [30, 31]. Artemisinin and its derivatives are considered to interact with haem/iron in the parasite food vacuole to generate free radicals which are toxic to parasites [25, 26]. Free radicals generated in the parasite food vacuole promote haem-catalyzed oxidation of the vacuolar membrane, which leads ultimately to vacuole rupture and parasite autodigestion [32]. Artemisinin and its derivatives have potent anti-malarial activity, but two drawbacks. First, their elimination half-life is short, about 45 min [28, 33]. Second, unlike other oxidant drugs, artemisinin cannot be cyclically oxidized and reduced [29, 30]. Therefore, one drug molecule can generate only one free radical [34]. In vitro [25] and in vivo [35] studies showed that this anti-malarial effect of artemisinin was reduced by vitamin E treatment. The additive effect of combination treatment as shown in this study might be caused by the lowering effect of probucol on host plasma vitamin E concentration, which prevented the elimination of free radicals generated by DHA.

Delayed haemolysis after treatment with intravenous artesunate has been detected in prospective studies in 7–21 % of patients with severe malaria [36, 37]. Especially in travellers from non-endemic regions, cases of post-artesunate delayed haemolysis are one of the more frequent events [36, 38, 39]. Although parasitaemia of DHA single treated mice was almost cleared, haemoglobin concentration was so low on 24 dpi (Fig. 6b, c). On the other hand, probucol and DHA combined treated mice showed significantly higher haemoglobin concentration and same degree of parasitaemia compared with DHA single treated mice on 24 dpi. Probucol treatment might inhibit the haemolytic effect of DHA or the down-regulation of parasitaemia development by probucol treatment might moderate anaemia. This effect of probucol may be beneficial for travellers as a prophylactic drug.

## Conclusions

This study demonstrated that probucol impacts on *P. yoelii* XL-17 infection by oxidative stress through the induction of host plasma vitamin E deficiency. Moreover, the effect of DHA on *P. yoelii* XL-17 infection was enhanced by probucol treatment. This finding indicates that probucol might be a candidate for a prophylactic treatment drug to enhance the effect of DHA.

## Abbreviations

ACT: Artemisinin-based combination therapy
DHA: Dihydroartemisinin
iRBC: Infected red blood cells
WHO: World Health Organization
